# Cleanliness of Canal Walls following Gutta-Percha Removal with Hand Files, RaCe and RaCe plus XP-Endo Finisher Instruments: A Photographic *in Vitro* Analysis 

**DOI:** 10.22037/iej.2017.47

**Published:** 2017

**Authors:** Kasra Karamifar, Neda Mehrasa, Pouyan Pardis, Mohammad Ali Saghiri

**Affiliations:** a *Department of Endodontics, Dental School, Islamic Azad University, Shiraz Branch, Shiraz, Iran; *; b * Wisconsin Institute for Medical Research, University of Wisconsin School of Medicine and Public Health, Madison, WI, USA*

**Keywords:** Gutta-Percha Removal, RaCe, Root Canal Retreatment, XP-endo Finisher

## Abstract

**Introduction:**

Gutta-percha must be removed from the root canal space during retreatment to ensure a more favorable outcome. The aim of this study was to compare the efficacy of hand instruments, RaCe and RaCe plus XP-endo finisher instruments in removal of gutta-percha from root canal walls during retreatment.

**Methods and Materials:**

Thirty single-rooted premolars were prepared, obturated, and divided into three groups according to retreatment method; in group 1, retreatment was carried out by hand instruments, while in groups 2 and 3 retreatment was done using RaCe rotary files alone or accompanied by XP-endo finisher instruments, respectively. After retreatment, teeth were sectioned longitudinally and photographic images were taken. The amount of remaining gutta-percha in coronal, middle and apical thirds was quantified using Image J software. The two-way ANOVA and post hoc Tukey’s tests were used to analyze data. The level of significance was set at 0.05.

**Results:**

RaCe cleaned the apical third significantly better than hand instrumentation. In the coronal third, RaCe+XP-endo finisher was more effective than RaCe. RaCe+XP-endo finisher was more effective than hand instrumentation in the entire root canal. The amount of remaining gutta-percha was the least in the apical part and increased toward the coronal part with the use of XP-endo finisher (*P*<0.05).

**Conclusion:**

Rotary instrumentation was more effective in removing gutta-percha from the canal walls. Furthermore, use of XP-endo finisher file resulted in cleaner canal walls and was more effective in removing gutta-percha from the coronal toward the apical part of the canal.

## Introduction

Complete removal of gutta-percha (GP) from the root canal system is a major goal in retreatment and it can be time-consuming and challenging [1-3]. Retreatment is recommended in order to re-establish healthy periapical tissues after inefficient treatment or re-infection of the obturated root canal system because of coronal or apical leakage [4, 5]. It requires regaining access to the entire root canal system through removal of the original root canal filling, further cleaning and disinfection and finally re-obturation [6]. Necrotic tissue or bacteria, covered by remaining GP or sealer, may be responsible for periapical inflammation or pain [7]. Residual bacteria have to be uncovered through removal of as much obturation material as possible. This enables thorough chemo-mechanical re-instrumentation and re-disinfection of the root canal system [8]. The primary goal of root canal retreatment is to stop the infectious process through the removal of filling material, debris and microorganisms that cause apical periodontitis [9, 10]. 

**Figure 1:**
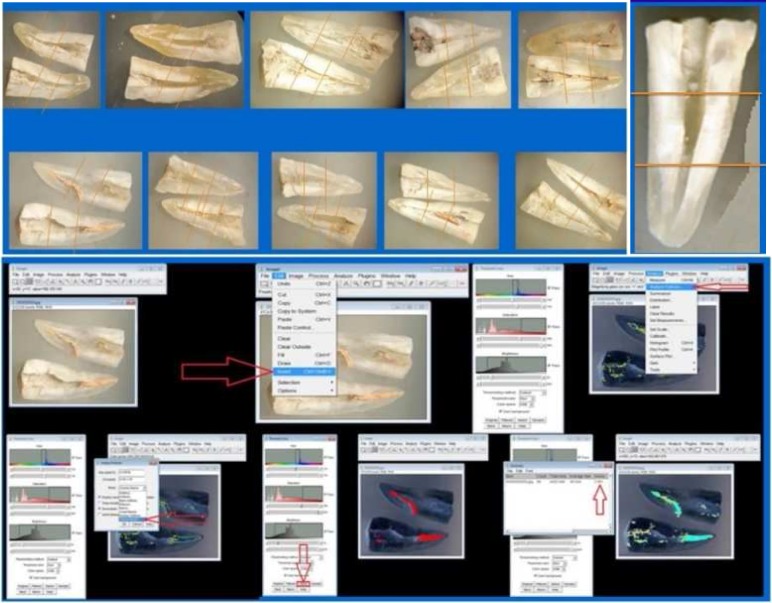
A) Sectioning and dividing the teeth. The surface area of each sample can be seen; B) Sectioning and dividing surface area into three parts, (Coronal, Middle and Apical); C) Defining and measuring the surface area of the canal and remaining gutta-percha

Many different instrumentation motions [11] and devices are available for removing GP, including hand files, nickel-titanium (NiTi) rotary instruments [12-14], ultrasonic devices [15, 16] and lasers [17, 18]. However, none of these techniques are fully effective in removing the filling materials [4, 5, 14, 19].

XP-endo finisher (FKG Dentaire SA, La Chaux-de-Fonds, Switzerland) is a rather new endodontic instrument which is introduced in an attempt to gain access to unreachable canal areas. It can be used after any root canal instrumentation to accomplish better canal cleanliness while conserving root canal dentin. The tip size is #25 with zero taper [20, 21]. 

The aim of this *in vitro* study was to compare the efficacy of hand instruments, RaCe, and RaCe + XP-Endo finisher, in removing gutta-percha from the walls of endodontically treated canal.

## Materials and Methods

***Sample preparation***

This experimental *in vitro *study was performed after approval by Ethics Committee of Dental School, Shiraz Branch, Islamic Azad University (approval ID: 95/736). Thirty single-rooted straight premolars with fully formed apices and no calcifications or internal resorption were used. The specimens were immersed in 0.5% chloramine-T solution (Merck, Darmstadt, Germany) for 48 h for disinfection and then stored in 4^°^C distilled water. Soft tissues and calculi were removed mechanically from the root surfaces with a periodontal scaler. Access cavities were prepared. The size of the minor foramen was controlled by inserting a #10 K-file to the working length. To standardize the samples, the crowns were removed to leave a 16-mm root. The teeth were randomly assigned to 3 groups based on the retreatment techniques used. A single operator prepared all the root canals.

***Cleaning and shaping***

In group 1 (*n*=10), ten samples were prepared according to the following description. The apical part was prepared up to a #35 stainless steel K-file (Mani, Matsutain Seisakusho Co., Tochigi-Ken, Japan), and the middle and cervical thirds were flared and refined up to #70 K-file with one-mm increments. At each instrument change, the root canal was irrigated with 2 mL of 2.5% NaOCl solution delivered using a syringe with a 27-gauge needle (Ultradent, South Jordan, UT, USA). A final rinse with 5 mL of distilled water was used to remove the previously used solutions. The root canals were dried using paper points (Gapadent Co., Ltd., Korea).

In group 2 and 3 (*n*=10), RaCe instruments (FKG Dentaire, La-Chaux-de-Fonds, Switzerland) were used according to the manufacturer’s instruction (at speed of 600 rpm and torque of 150 g/cm) set on torque-controlled motor (X-Smart, Dentsply Maillefer, Ballaigues, Switzerland). The apical part was prepared up to 35/0.04.

The obturation protocol were the same in all groups. The root canals were filled with lateral condensation of GP (Gapadent Co., Ltd., Korea) and epoxy resin sealer (AH-26, Dentsply, De-Trey, Konstanz, Germany). The sealer was mixed according to the manufacturer’s instructions, and #35 gutta-percha master cones were coated with sealer and placed in the root canal to the working length. After cutting back the fillings, the root canals were sealed with Coltosol (Asia Chemi Teb. Co., Tehran, Iran) temporary filling, and the teeth were stored at 37^°^C under 100% humidity for 2 weeks [19] to allow the sealer to set to the maximum extent.

**Figure 2:**
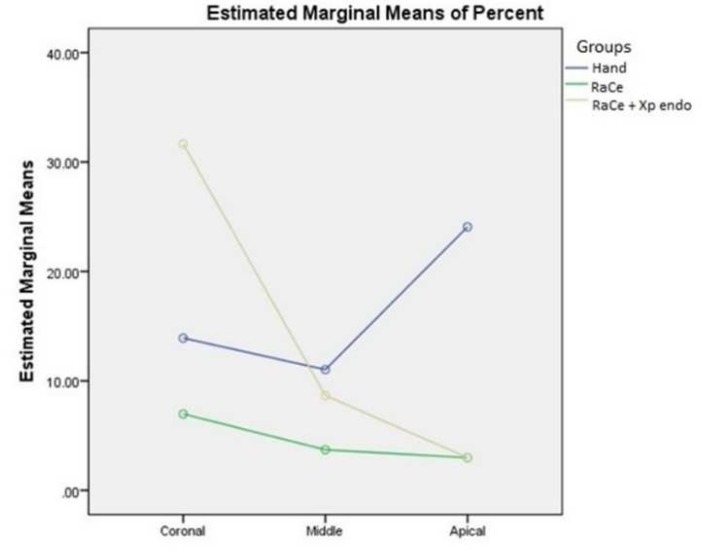
The difference between the canal thirds in each group. The amount and percentage of gutta-percha removal from coronal toward apical was greater when using RaCe+Xp endo

**Figure 3:**
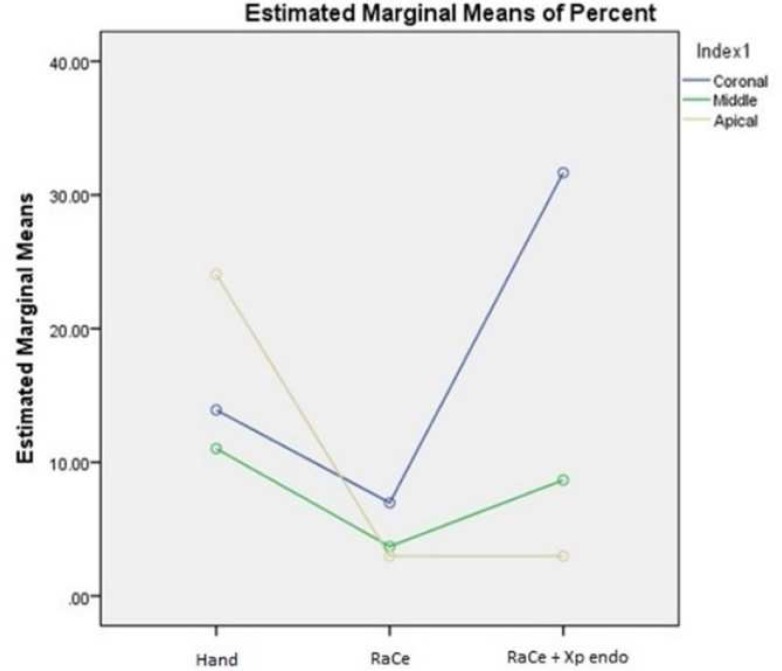
The difference between the three groups in each part of the canal. RaCe+Xp endo removed guatta-percha better in the apical third than hand instrumentation

***Retreatment***

In group 1, the temporary fillings were removed. During retreatment, the root canals were irrigated with 2 mL of 2.5% NaOCl solution at each instrument change. The coronal part of GP was penetrated with a #1 Gates-Glidden drill (Mani, Matsutain Seisakusho Co., Tochigi-Ken, Japan). The apical part was enlarged up to a #40 stainless steel H-file (Mani, Matsutain Seisakusho Co., Tochigi-Ken, Japan) and the canal was prepared in 1-mm increments up to #80. No solvent was used for softening the gutta-percha.

In group 2, the same procedure was used to penetrate gutta-percha. The apical part was prepared up to 40/0.04 RaCe rotary files. The other parts of the sample preparation were the same as those in group 1. 

In group 3, samples were prepared using RaCe instruments similar to group 2 (final preparation size 40/0.04). Afterwards XP-endo finisher instrument was used in each canal which was filled with 2.5% NaOCl for 1 min (at speed of 800 rpm and torque of 100 g/cm).

***Evaluation of residual material***

To evaluate the residual filling material, the teeth were grooved buccolingually using a double-sided diamond disk (KG Sorensen, Barueri, Brazil) and sectioned longitudinally using an Ochsenbein chisel. Both root halves were photographed under a stereomicroscope (Olympus BX43, Olympus Co., Tokyo, Japan). Digital images were obtained under ×6 magnification from both halves using a stereomicroscope attached to a digital camera (Nikon D90, Nikon Corporation, Tokyo, Japan) and transferred to a computer. On these digital images, the remaining filling material was calculated as a percentage (Figure 1).

The area of residual filling on all root canal surfaces (total area) and in each root canal third (cervical, middle and apical) was measured. The percentage of residual filling material in the root canal walls was calculated using the following equation: (area of the remnant × 100/area of the root canal

***Image analysis***

The images were transferred to image analysis software (ImageJ software, National Institutes of Health, Bethesda, MD, USA) to measure the areas of residual filling material and root canal walls (Figure 1). 

***Data analysis***

Data were analyzed at 95% confidence level (*P*<0.05). Statistical analysis was performed with Statistical Package for Social Science (SPSS, version 18.0, SPSS, Chicago, IL, USA). Two-way ANOVA and post hoc Tukey’s tests were used to analyze data.

## Results

The results of the amount of remaining gutta-percha are presented in Table 1. In the apical third, RaCe proved significantly better than hand instrumentation. In the coronal third, XP-endo finisher was significantly better than RaCe. XP-endo finisher was significantly better than hand instrumentation in the entire root canal. The amount of remaining GP was the least in the apical part and increased toward the coronal part with the use of XP-endo finisher (*P*<0.05) (Figures 2 and 3). 

## Discussion

This *in vitro* study assessed the efficacy of the Xp-endo finisher file on the removal of gutta-percha from canal walls after retreatment. This instrument was able to minimize the remaining obturating materials on canal walls when used after the rotary instrumentation as a finisher file. The performance of the Xp-endo finisher file was increased from the coronal toward the apical third. 

After root canal retreatment the amount of residual obturation materials in the canal is minimized when canal enlargement during the retreatment procedure is larger than the enlargement size performed before root canal obturation [22, 23]. Therefore, the retreatment procedure was completed with an instrument one size larger (40/0.04) than enlargement size during primary preparation (35/0.04). In addition, standardization of additional instrumentation was made and performed using a #40/0.04 apical diameter for groups retreated with rotary files and a #40 apical diameter and 0.05 taper for group retreated with hand instruments [14, 24]. The performance of 40/0.04 RaCe was consistent with the findings of other studies that have evaluated its shaping ability [25, 26], and this may be related to its design. These instruments have a simple triangular cross-section, high cutting ability, alternating cutting edges, and bullet shape tip design which favors instrument penetration into the filling material. The flute area of these instruments allows coronal extrusion of filling materials [27].

Although complete removal of GP in some samples was achieved, which was consistent with some previous studies [19, 24], both hand and rotary instrumentation techniques failed to remove all the filling material from the canal walls [3-5, 28, 29]. More residual filling material was found in the apical third compared to the middle [1, 30, 31] and in the coronal third compared to middle third. However, in XP-endo finisher group this did not happen. In the present study, significantly less debris remained in the apical part compared to the middle third when XP-endo finisher was used (*P*<0.05) and also significantly less debris remained in the middle part compared to the coronal part (*P*<0.05). There were no significant differences between different thirds in other groups (*P*>0.05). The relatively higher residual filling material in the coronal third might be due to the fact that larger sizes of Gates-Glidden drills were not used, which is consistent with some previous studies [11]. Furthermore XP-endo finisher was significantly more effective in root canal cleaning compared to RaCe from the coronal toward the apical third. Race was significantly more effective than hand instrumentation regarding gutta-percha removal in the apical third (*P*<0.05). The efficacy of the XP-endo finisher in removing gutta-percha from the canal walls might be attributed to its metallurgy and elliptical movement in the canal. This movement and design can help reaching inaccessible parts of the canal. While XP-endo finisher rotates a curved bulb is formed which can expand its extent 6 mm in diameter when the file tip is squeezed or 100-times of a corresponding sized file [20]. Residual filling material has been assessed using many techniques including radiography [32, 33], longitudinal sectioning prior to microscopic or photographic analysis [3, 34, 35] and micro-computed tomography scanning (µ-CT) [14, 29, 36]. Similar to previous studies [3, 34], the roots were split vertically to evaluate the presence of root filling material remnants under stereomicroscope. This method offers advantages over other techniques because it is easy to use and the distance between the object and the device is constant, enabling image standardization. This methodology has been shown to be more effective than radiographic techniques in investigating remaining filling material [33, 37]. In this study, we used vertical splitting to obtain images for observation after retreatment, which is a well-established method [24, 38]. 

Removal of filling materials during retreatment allows effective action of instruments and irrigating solutions on debris and microorganisms responsible for apical periodontitis [19, 34, 38-41]. XP-endo finisher was more effective in removing gutta-percha from the coronal toward the apical part of the canal (*P*<0.05). Optimal cleaning while preserving dentin is the goal of retreatment. This may be due to the movement in the canal and the way it expands in the canal. The way it moves may help it touch the canal in almost impossible-to-reach areas. In this way it is able touch biofilm-covered areas and the irrigants may be more effective against bacteria and their biofilm, which should be evaluated by further studies. 

**Table 1:** Mean (SD) of the amount of remaining gutta-perch in tested groups

**Groups**	**Hand**	**RaCe**	**RaCe+Xp-endo**
**Coronal**	14208.5750 (4030.32052)	20775.1250 (5488.19628)	24109.0250 (7945.08047)
**Middle**	7730.0500 (2530.56903)	2794.9750 (1039.88307)	2951.5000 (1040.24837)
**Apical**	7639.0690 (3050.88816)	2973.6250 (1643.19378)	957.6250 (379.22091)

ImageJ is a Java-based, open source software program and fulfills most routine image processing and analysis requirements. ImageJ has been used in some previous endodontic studies [42, 43]. Some of the advantages of this software are accuracy, co-localization, support for a wide number of standard image file formats, and the ability to run on different platforms [44]. This method of evaluation was non-destructive; therefore, no intervention existed in evaluating the percentages of debris.

Most studies in the literature standardize the length of the teeth by sectioning the crowns [34, 38, 45]. In the current study, standardization was achieved by partial removal of crowns to attain similar working length in all the samples. In this study solvents were not used to limit the evaluation process to the efficacy of instruments on the outcome of gutta-percha removal. 

Rotary instrumentation was more effective in removing gutta-percha from the canal walls than hand instrumentation, which is consistent with some other studies. NiTi instruments are more efficient than hand instrumentation; they reduce clinical time and operator and patient fatigue [40].

## Conclusion

It can be concluded that under the limitations of this study rotary instrumentation was more effective in removing gutta-percha from the canal walls. Furthermore, use of XP-endo finisher file resulted in cleaner canal walls when used as a finisher file since all rotaries make round shapes. Unlike other rotary instruments, XP-endo finisher was more effective in removing gutta-percha from the coronal toward the apical part of the canal.
